# Advances in immunotherapy modalities for atherosclerosis

**DOI:** 10.3389/fphar.2022.1079185

**Published:** 2023-01-10

**Authors:** Qingwen Meng, Huajiang Liu, Jinteng Liu, Yangyang Pang, Qibing Liu

**Affiliations:** ^1^ Department of Pharmacy, The First Affiliated Hospital of Hainan Medical University, Haikou, China; ^2^ Deparment of Cardiovascular, The First Affiliated Hospital of Hainan Medical University, Haikou, China; ^3^ Hainan Provincial Key Laboratory of Tropical Brain Research and Transformation, Hainan Medical University, Haikou, China; ^4^ School of Basic Medicine and Life Sciences, Hainan Medical University, Haikou, China

**Keywords:** atherosclerosis, innate immunity, adaptive immunity, immunotherapy, immunological targets

## Abstract

Cardiovascular disease (CVD) is one of the leading causes of death worldwide. Atherosclerosis is the pathological basis of atherosclerotic cardiovascular disease (ASCVD). Atherosclerosis is now understood to be a long-term immune-mediated inflammatory condition brought on by a complicated chain of factors, including endothelial dysfunction, lipid deposits in the artery wall, and monocyte-derived macrophage infiltration, in which both innate immunity and adaptive immunity play an indispensable role. Recent studies have shown that atherosclerosis can be alleviated by inducing a protective immune response through certain auto-antigens or exogenous antigens. Some clinical trials have also demonstrated that atherosclerotic is associated with the presence of immune cells and immune factors in the body. Therefore, immunotherapy is expected to be a new preventive and curative measure for atherosclerosis. In this review, we provide a summary overview of recent progress in the research of immune mechanisms of atherosclerosis and targeted therapeutic pathways.

## Introduction

Cardiovascular disease (CVD) continues to be the leading cause of morbidity and mortality worldwide, despite major improvements in treatment and outcomes (Roth et al., 2020). Atherosclerotic cardiovascular disease (ASCVD) refers to clinically diagnosed atherosclerotic diseases, including coronary artery disease (CAD), stroke, and peripheral arterial disease. Atherosclerosis is the pathological basis of ASCVD. The illness usually advances slowly, with lipid-laden plaques forming in large and middle-sized arteries. It may go unnoticed for years before thrombus formation, which obstructs arteries and damages nearby tissues due to ischemia and plaque erosion or rupture. Current drug treatment for ASCVD mainly consists of statins, anti-platelet drugs. Large residual risk still exists in the treatment of ASCVD despite the significant reduction in ASVCD demonstrated in large-scale clinical studies using statins Baigent et al., 2005. This residual risk may be due to the involvement of immune cell-mediated inflammatory responses involved in the pathogenesis of ASCVD. There is growing evidence that the immune response is involved in the onset and progression of atherosclerosis (Williams et al., 2019; Wolf and Ley, 2019). Targeted immune mechanisms are expected to be an effective treatment for ASCVD (Ley, 2016). It has been found that interactions between innate and adaptive immune cells can lead to immune regulation and activation of cytokines and chemokines, which influence the development of atherosclerosis in experimental models (Williams et al., 2019; Wolf and Ley, 2019).

### Immune mechanisms of atherosclerosis

**FIGURE 1:**
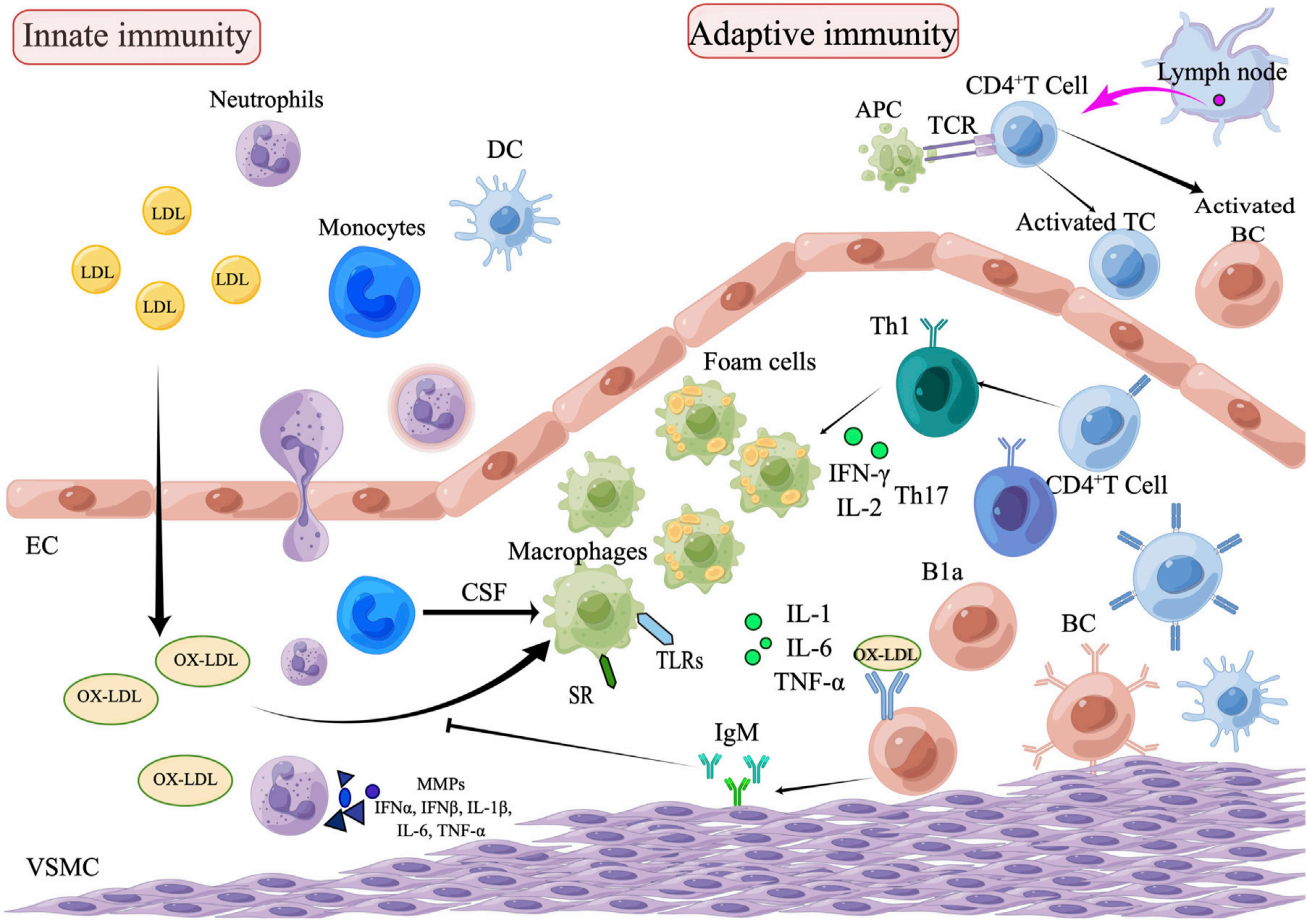
The role of innate and adaptive immunity in the development of atherosclerosis. The process of atherosclerosis is brought on by the buildup of heat shock proteins and modified ox-LDL in the intima of the arterial wall, which is exacerbated by the invasion of innate immune cells such as monocytes, eosinophils, and neutrophils. Monocytes are transformed into macrophages, which remove ox-LDL by binding to scavenger receptors and are further transformed into foam cells. Subsequently, adaptive immune cells, including Th1and Th17, enter the plaque lesion and produce inflammatory cytokines (IL-1, IL-6, and TNF-α) which exacerbate the pathophysiology of atherosclerosis. APCs arrive *via* lymphatic vessels to present homologous peptides on MHC molecules to naive T cells, which attract adaptive T cell and B cell responses. B1a cells can be stimulated by IL-5 to produce oxygen LDL-specific natural IgM antibodies, which block oxygen LDL absorption and production of foam cells, thus achieving an anti-atherosclerotic effect. Natural IgM has also been demonstrated to facilitate the clearance of apoptotic cells (EC, endothelial cells; VSMC, vascular smooth muscle cell; DC, dendritic cell; TC, T lymphocytes; BC, B lymphocytes; SR, scavenger receptor; B1a, B1a lymphocytes; CSF, colony stimulating factors; APCs, Antigen-presenting cells).

Atherosclerosis is a long-term, lipid-driven, and immune-mediated inflammatory condition. Modified lipoproteins, subendothelial deposits and dysfunctional endothelial cells (ECs) behave as damage-associated molecular patterns that activate immune receptors, promote immune cell migration, and result in vascular inflammation during the early stages of atherosclerosis. Monocytes mature into macrophages stimulated by colony-stimulating factors (CSF). Macrophages upregulate pattern-recognition receptors (PRRs), including toll like receptors (TLRs) and scavenger receptors (SRs). Activation of the TLRs pathway leads to an inflammatory response, and SRs lead to the formation of foam cells by regulating oxidative LDL (ox-LDL), the foam cells form atherosclerosis plaques. The smooth muscle cells, macrophages and foam cells in the plaque undergo apoptosis and necrosis, then forming a necrotic core (Legein et al., 2013). By delivering antigens to T lymphocytes, dendritic cells (DCs) act as a bridge between the innate and adaptive arms of the immune response. T and B lymphocytes are involved in the atherosclerosis process by regulating the inflammatory response (Ketelhuth and Hansson, 2016; Sage et al., 2019) (Figure 1).

### Innate immunity and atherosclerosis

Innate immunity, also known as intrinsic immunity, serves as the body’s first line of defense and is a rapid nonspecific response. It is mainly composed of monocytes, macrophages and DCs. It is mediated by the generation of cytokines and chemokines, triggering the complement cascade and phagocytosis. In the physiological state, monolayer endothelial cells are closely lined with various connections on the surface of blood vessels to form the endovascular membrane, playing an important natural barrier role. Various damage factors such as free radicals, lipid accumulation, and abnormal blood flow lead to endothelial cell activation, endothelial dysfunction, and increased vascular permeability. LDL deposited under the vascular lining is oxidized to ox-LDL, thus activating ECs to express adherent molecules, attracting monocytes to migrate to the endothelium to differentiate into macrophages, engulfing ox-LDL through scavenger receptors to form foam cells, and finally becoming the atherosclerotic plaque (Orekhov and Sobenin, 2019; Poznyak et al., 2020). Endothelial cell activation secretes and releases vascular cell adhesion molecule 1(VCAM-1), intercellular adhesion molecule 1(ICAM-1), and chemokine ligand 16(CXCL16) (Charo and Ransohoff, 2006; Poznyak et al., 2020). Production of these cytokines is a key step for monocytes and neutrophils to roll along the vascular surface and adhere to the activated endothelium. Monocyte chemotactic protein 1 (MCP-1) further induces adherent monocytes to infiltrate the subintima of the aortic wall and differentiate into macrophages, causing the corresponding endothelial-activated intrinsic immune response (Deshmane et al., 2009). Intraplaque macrophages are mainly divided into M1 and M2 types, and their differentiation is stimulated by local oxidative LDL and other stimuli, which play different roles in atherosclerosis formation (Leitinger and Schulman, 2013). In recent years, with the development and rapid progress of single-cell technologies such as cytometry by time of flight and single-cell RNA sequencing, macrophages have been classified into several three types, resident-like, pro-inflammatory and anti-inflammatory foamy TREM2^hi^ macrophages. The study found that resident-like macrophages, which resemble the M2 phenotype, multiply and participate in endocytosis. As the primary source of inflammation in the lesion, inflammatory macrophages are linked to the M1 phenotype. The foamy lipid macrophages known as TREM2^hi^ macrophages aggregate in the plaque endothelium and its necrotic core. They also appear to have an M2-like behavior (Willemsen and de Winther, 2020; Zernecke et al., 2020). DCs play an antigen-presenting role in atherosclerosis plaque formation. Ox-LDL stimulates ECs to produce granulocyte-macrophage colony-stimulating factor (GM-CSF), which increases the adhesion and migration of DCs, thereby regulating the number of DCs in the plaque (Gil-Pulido and Zernecke, 2017).

### Adaptive immunity and atherosclerosis

Adaptive immunity activates the immune response through T and B-cell specific receptor to activate the immune response. There are two types of T cells: CD4^+^ and CD8^+^ T cells. Th1, Th2, Th17 and CD4^+^ regulatory T cells (Treg) are subtypes of CD4^+^ T cells. Intraplaque macrophages and DCs can act as antigen-presenting cells (APCs) which phagocytose antigens and present them to T cells, forming the APC-T cells axis (Koltsova et al., 2012). Th1 cells secrete pro-inflammatory factors such as IFN-γ and tumor necrosis factor alpha **(**TNF-α**)**, which initiate or promote atherosclerosis inflammatory responses and increase plaque instability by activating monocyte-macrophages and DCs and affecting Treg stability (Kyaw and Bobik, 2020). The main cytokine of Th2 cells is IL-4, which binds to the IL-4 receptor in T cells and promotes the expression of the transcription factor GATA-3, however, it is not clear whether IL-4 has a proatherogenic or protective effect on atherosclerotic plaques. However, other cytokines associated with Th2 cells, such as IL-5 and IL-13, have been shown to be protective against atherosclerosis. IL-13 can reduce the expression of VCAM1, resulting in reduced macrophage infiltration in plaques (Ou et al., 2018; Saigusa et al., 2020). Th17 cells have also been found to be involved in atherosclerosis formation, but their role is controversial in academic circles (Taleb and Tedgui, 2018; Nordlohne and von Vietinghoff, 2019). Treg is a subset of T cells that control autoimmune reactivity *in vivo*. Treg can inhibit the inflammatory response within the plaque and plays a protective role (Ou et al., 2018). Treg cells suppress the growth of pro-inflammatory effector T cells and secrete IL-10 and TGF-β to exert their atheroprotective effects (Robertson et al., 2003; Ait-Oufella et al., 2006). Treg cells combat effector T cells and extra-atheroma impacts the cholesterol metabolism to exercise their anti-atherosclerotic action (Mor et al., 2006).

Initial studies showed that the role of B lymphocytes in atherosclerosis tended to be protective (Porsch et al., 2021), but after the discovery of the presence of B cell subtypes B1 and B2 cells, there is still controversy regarding the specific role of different subtypes of B cells in atherosclerosis. In mice, B1 and B2 are the two major subtypes of B cells. B1 cells have a potent anti-atherosclerosis effect by secreting IgM antibodies to recognize the surface antigenic determinants of apoptotic cells and ox-LDL, thereby inhibiting foam cell formation and blocking the uptake of ox-LDL. In addition, the concentration of anti-oxLDL specific IgM antibody was found to be negatively correlated with adverse clinical manifestations of ASCVD (Tsiantoulas et al., 2014). Most studies seem to indicate a pro-atherosclerosis effect on B2 cells, but the action mechanism remains unclear (Centa et al., 2019).

## Immunotherapy in atherosclerosis

**FIGURE 2:**
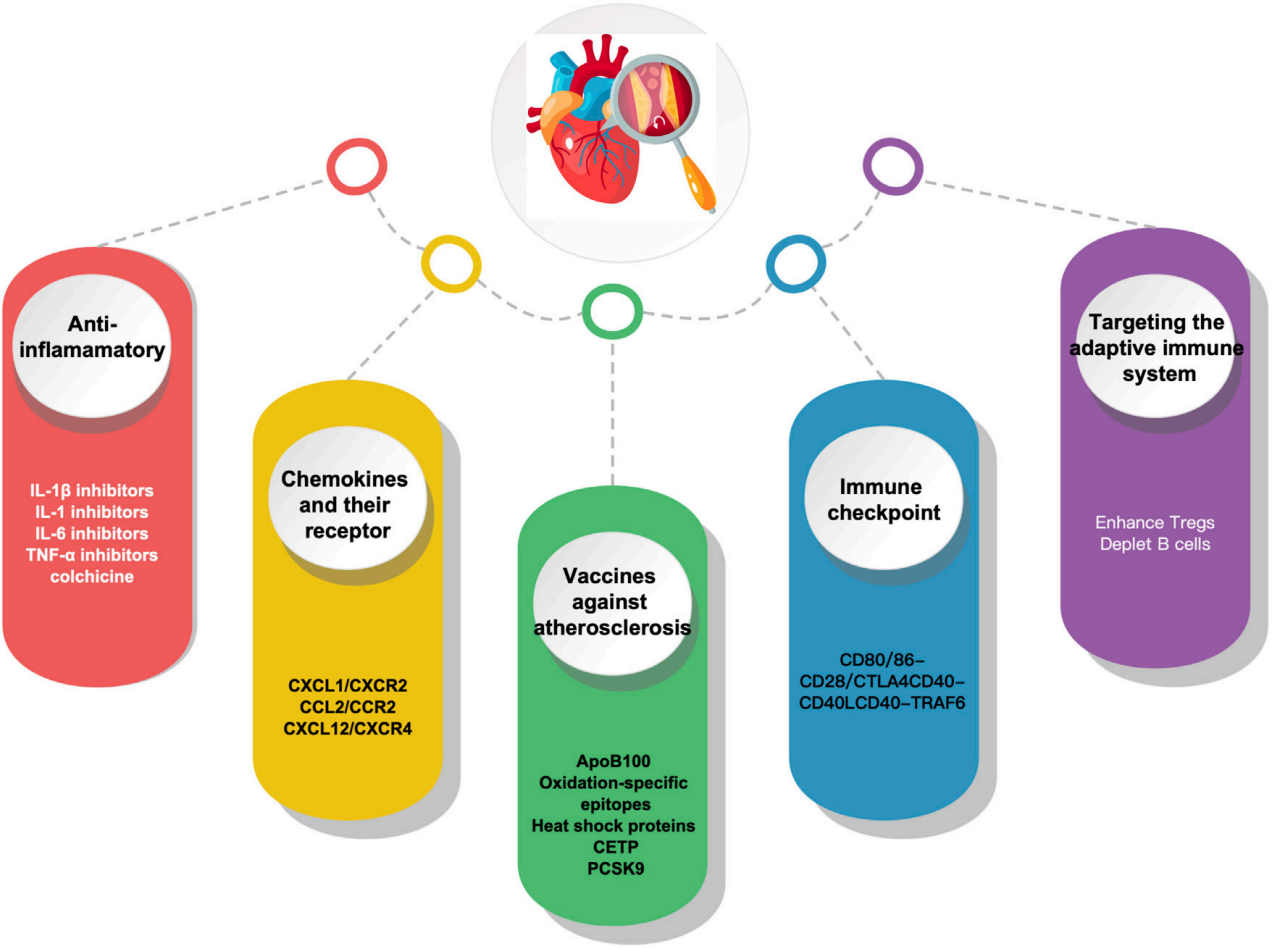
Immunotherapy in atherosclerosis.

Understanding the immunopathological process implicated in atherosclerosis can provide fresh perspectives on ASCVD prevention and treatment. Given the key role immune activation plays in atherosclerosis, immunotherapy appears to be a potential strategy. With the progress of research on the immune mechanism of atherosclerosis, it has become possible to combat atherosclerosis through immunomodulation-related targets, and the main immunotherapeutic measures currently available include the following anti-inflammatory, chemokines and their receptors, Vaccines, immune checkpoints and targeting adaptive immunity (Figure 2).

## Anti-inflammatory therapies

### Interleukin-1 beta (IL-1β) inhibitors

The key role played by the pro-inflammatory cytokine IL-1β in the atherosclerotic process (Ross, 1999). This includes promoting monocyte and leukocyte adhesion to endothelial cells, inducing procoagulant activity, and promoting the growth of vascular smooth muscle cells (VSMC). IL-1β is an upstream factor in the inflammatory waterfall chain. This increases the expression of IL-1β canonical proinflammatory mediators like IL-6, IL-8, IL-1α, TNF-α, adhesion molecules, and MCP-1 (Bermúdez et al., 2018). NLRP3 inflammasome plays an important role in foam cell death and chronic vascular inflammation. The superstructure of NLRP3 is expressed in some immune cells and when this inflammatory vesicle is activated, it eventually produces IL-1β, IL-18 and IL-33. A study reported that inhibition of NLRP3/IL-1β pathway significantly reduced the atherosclerosis plaque size and inflammatory response (Libby, 2017). Canakinumab is a monoclonal antibody to human IL-1β that binds specifically to human IL-1β, preventing it from binding to IL-1R1 and thus preventing IL-1β from exerting its biological effects (Rondeau et al., 2015). The CANTOS study was designed as a randomized, double-blind, placebo-controlled international large-scale multicenter clinical trial enrolling 10,061 patients with a history of myocardial infarction (MI) with elevated (>2 mg/L) hypersensitivity C-reactive protein (hsCRP) from 39 countries. All patients were randomized to four groups based on standard drug therapy: a placebo group and three Canakinumab (50 mg, 150 mg, and 300 mg) groups, in which Canakinumab was administered subcutaneously for every 3 months with a median follow-up period of 3.7 years. The results showed a 7%, 15% and 14% reduction in the risk of cardiovascular events as the primary endpoint in the three Canakinumab treatment groups as compared with the placebo group (Ridker et al., 2017). The CANTOS study confirmed that canakinumab can further reduce the incidence of adverse cardiovascular events in patients with myocardial infarction in addition to lipid-lowering drug therapy and provides direct evidence for the inflammatory hypothesis of atherosclerosis.

### IL-1 inhibitors

The biological cascades of IL-1 are blocked by the recombinant human IL-1 receptor antagonist anakinra, which is often used to lower systemic inflammatory reactions. Study showed that different doses of anakinra could reduce plaque in the aortic arch, suppress inflammatory factor expression, and lowered CD68^+^ macrophage infiltration in visceral fat in ApoE^−/−^ mice, suggesting that anakinra may be a helpful supplement to the standard treatment plan to lower the lingering cardiovascular risk (Ku et al., 2022). The MRC-ILA-HEART study randomized 182 individuals with non-ST elevation acute coronary syndrome (ACS) < 48 h into an anakinra group or a placebo group, finding that the hsCRP vAUC value was reduced by nearly 50% in anakinra group versus placebo group in the first 7 days. On day 14, the hsCRP level in anakinra group was still significantly lower than that in placebo group. However, 16 days after discontinuation of the injection, the hsCRP level in anakinra group increased again. The incidence of major adverse cardiovascular events in anakinra group was still high after 1 year (Morton et al., 2015), suggesting that anakinra could improve the short-term vascular inflammatory response, though more research is required to support the use of anakinra to treat atherosclerosis.

In another VCU-ART study on the improvement of myocardial remodeling by anakinra after acute myocardial infarction, this small study enrolled 10 patients with ST-segment elevation myocardial infarction and randomized to anakinra 100 mg/day or placebo. Cardiac magnetic resonance (CMR) at 10 days and 14 days of pharmacological intervention compared the ratio of LV end-systolic volume, and the results showed that the anakinra treatment group reduced LVESVi and reversed left ventricular remodeling in both CMR and cardiac echocardiography. VCU-ART study has shown blocking IL-1 with anakinra in patients with ST-segment elevation AMI is safe and has a favorable effect on left ventricular remodeling (Abbate et al., 2010). Therefore, further large-scale clinical studies on anakinra in ASCVD are needed in the future.

### IL-6 inhibitors

IL-6 is a circulating cytokine that is secreted by many different cells, including macrophages, monocytes, and fibroblasts and endothelial cells (Ridker and Rane, 2021). IL-6 is a powerful inducer of Th17 cells and a pro-inflammatory cytokine. A downstream pro-inflammatory condition brought on by IL-6 signaling causes a rise in hsCRP, fibrinogen, and other acute phase reactants. In ApoE^−/−^ mice, systemic administration of IL-6 caused an increase in atherogenesis, confirming the idea that IL-6 is a pro-atherosclerotic cytokine (Huber et al., 1999). Studies have shown that plasma levels of IL-6 are increased in unstable angina in patients with CAD (Cheng et al., 2008).

Tocilizumab is an anti-IL-6 receptor monoclonal antibody. In rheumatoid arthritis patients, tocilizumab has been demonstrated to diminish articular inflammation and promote disease remission (Favalli, 2020). In a two-center, double-blind, placebo-controlled trial, 117 patients with non-ST-segment elevation MI were randomized into two groups who were given a single dose of placebo or tocilizumab before coronary angiography. The main indicators tested were hsCRP and high-sensitivity TnT (hsTnT). The median AUC of hsCRP and hsTnT during hospitalization was 2.1-fold and 1.7-fold higher in placebo group than that in tocilizumab group respectively. In NSTEMI patients, tocilizumab reduced the inflammatory response and predominantly percutaneous coronary intervention (PCI)-related TnT release (Kleveland et al., 2016).

Zevacizumab (ziltivekimab) is an investigational narrow-spectrum fully human monoclonal antibody against IL-6 ligands. Unlike other clinically available IL-6 signaling inhibitors, zevacizumab was developed specifically for the treatment of atherosclerosis. RESCUE was a randomized double-blind phase 2 clinical trial conducted at 40 clinical centers in the United States. It had been designed to investigate whether zevacizumab could safely and effectively reduce biomarkers of inflammation and thrombosis in patients at high risk of CVD. The inclusion criteria were patients over 18 years of age with moderate-to-severe chronic kidney disease and hsCRP ≥2 mg/L. They were equally randomized to a subcutaneous placebo group and three (7.5 mg, 15 mg, and 30 mg) zevacizumab groups at a 4-week cycle, totaling 24 weeks. The primary outcome was the percentage change in hsCRP vs the baseline levels after 12 weeks of zevacizumab vs placebo treatment. The results showed that zevacizumab decreased the hsCRP level in a dose-dependent manner as compared with the placebo group (Ridker et al., 2021).

A large international cardiovascular outcomes trial called ZEUS was conducted in 2021 to compare ziltivekimab to placebo in approximately 6,000 patients with CKD and elevated hsCRP to formally determine whether a novel anti-inflammatory approach that directly reduces circulating IL-6 can reduce the incidence of cardiovascular events. While the primary endpoint of ZEUS is the occurrence of major adverse cardiovascular events including myocardial infarction, stroke and cardiovascular death, an important secondary objective of the trial is to address whether chronic IL-6 inhibition slows the progression of kidney disease (Ridker, 2021; Ridker and Rane, 2021). The ZEUS trial will provide strong support for the use of IL-6 in CVD.

### Tumor necrosis factor alpha (TNF-α) inhibitors

TNF-α is a pro-inflammatory cytokine that plays an important role in adaptive immunity and promoting atherosclerosis. Studies have shown that monocytes, macrophages, and smooth muscle cells can produce TNF-α. TNF has a key role in controlling inflammatory cascades. During atherosclerosis, TNF promotes an inflammatory cascade that occurs within the artery wall (McKellar et al., 2009). TNF has also been linked to causing endothelium damage by attracting immune cells like neutrophils, which can cause tissue damage (Quinn et al., 2008). TNF-α inhibitors, which are antibodies that lower inflammation in both atherosclerosis and autoimmune illnesses like rheumatoid arthritis, include infliximab, etanercept, and adalimumab (Jacobsson et al., 2005).

A study to determine the association between TNF inhibitors and risk of ASCVD in patients with rheumatoid arthritis, which included 2,101 patients with rheumatoid arthritis in a retrospective cohort study, showed a reduced risk of CHD in patients using TNF-α inhibitors and methotrexate (Bili et al., 2014). A systematic review and meta-analysis evaluating the relationship between anti-tumor necrosis factor (anti-TNF) therapy and cardiovascular events in patients with rheumatoid arthritis showed that cardiovascular events were related with a lower risk when anti-TNF medication was used (Barnabe et al., 2011). This data demonstrates that TNF has a significant pro-inflammatory role in atherosclerotic cardiovascular disease as well, and it raises the possibility that TNF suppression might help with cardiovascular prevention (Bäck and Hansson, 2015). In addition, more research is required to verify the atheroprotective effects of TNF suppression.

### Others

The COLCOT trial demonstrated for the first time the cardiovascular benefits of colchicine’s anti-inflammatory effects, reducing the risk of major cardiovascular events in patients after myocardial infarction by 23% (Tardif et al., 2019). The LoDoCo2 trial then confirmed that taking .5 mg of colchicine per day reduced the risk of major cardiovascular events by 31% in patients with chronic coronary heart disease (Fischer, 2021). The CIRT study was a double-blind randomized controlled trial enrolling 7,000 patients with CAD who had a previous myocardial infarction or type 2 diabetes/metabolic syndrome, with the study group receiving 15–20 mg of methotrexate per week and the control group receiving a placebo. The results of the study showed that methotrexate failed to reduce levels of IL-1, IL-6 and hsCRP. The incidence of elevated liver enzymes, hematocrit, and non-basal cell skin cancer was higher in the methotrexate group than in the control group (Ridker et al., 2019). (Table 1)

**TABLE 1:** The clinical experimental study of anti-inflammatory therapy for ASCVD.

Study name	Agent	Target	Patient cohort	Primary end point	Outcome	References
CANTOS	Canakinumab	IL-1β antibody	10,061 individuals with a history of MI and elevated plasma CRP levels	Nonfatal myocardial infarction, nonfatal stroke, or cardiovascular death	Independent of lipid-level lowering, anti-inflammatory therapy using canakinumab at a dose of 150 mg every 3 months significantly reduced the rate of recurrent cardiovascular events compared to placebo	Ridker et al. (2017)
COLCOT	Colchicine	Tubulin disruption; inhibition of inflammasome assembly and IL-1 release	4,745 patients with MI within 30 days before enrolment	Death from cardiovascular causes, resuscitated cardiac arrest, MI, stroke, or hospitalization for angina leading to coronary revascularization	Under standard care, colchicine .5 mg/day significantly reduced the risk of a first ischaemic cardiovascular event (23%) and total ischaemic cardiovascular event (34%)	Tardif et al. (2019)
LoDoCo2	Colchicine	Tubulin disruption; inhibition of inflammasome assembly and IL-1 release	5,522 patients with chronic coronary artery disease	Death from cardiovascular causes, spontaneous MI, ischaemic stroke or ischaemia driven coronary revascularization	Low-dose (.5 mg/day) colchicine in patients with acute myocardial infarction significantly reduces cardiovascular events during follow-up	Ridker et al. (2019)
MRC-IL1-HEART	Anakinra	IL-1 receptor antagonist	182 individuals with Non-ST elevation ACS <48 h	The hsCRP AUC value was reduced by nearly 50% in anakinra group versus placebo group in the first 7 days	hsCRP levels were lower after 14 days of anakinra medication; the risk of MACE was similar at 30 and 3 months but significantly higher at 1 year in the anakinra group compared to the placebo group	Morton et al. (2015)
Kleveland	Tocilizumab	IL-6	117 NSTEMI patients were randomized at a median of 2 days after the beginning of symptoms	The hsCRP AUC at 1–3 days of treatment initiation	Tocilizumab reduced hsCRP and hsTnT levels compared with placebo	Kleveland et al. (2016)
RESUE	Ziltivelimab	IL-6	264 patients with chronic kidney disease and hsCRP >2 mg/L	The primary outcome was the percentage change in hsCRP vs the baseline levels after 12 weeks of zevacizumab vs placebo treatment	Zevacizumab decreased the hsCRP level in a dose-dependent manner as compared with the placebo group	Ridker et al. (2021)
ZEUS	Ziltivelimab	IL-6	6,200 patients with chronic kidney disease and CRP ≥2 mg/L	The occurrence of major adverse cardiovascular events including myocardial infarction, stroke and cardiovascular death	Not yet completed	Ridker and Rane, (2021)
CIRT	Methotrexate	Purine metabolism inhibitor	4,786 patients with previous myocardial infarction or multivessel coronary disease who additionally had either type 2 diabetes or the metabolic syndrome	Nonfatal myocardial infarction, nonfatal stroke, or cardiovascular death	No benefit	Ridker et al. (2019)
——	TNF-α inhibitor	TNF-α	A retrospective cohort of 2,101 incident RA patients was established	Primary outcome was adjudicated incident CAD.	Use of TNF inhibitors and Lower Risk of Coronary Events in Patients with Rheumatoid Arthritis	Bili et al. (2014)

All the figures in this article.

## Chemokines and their receptors

Chemokines and their receptors are widely and significantly expressed in ECs, smooth muscle cells (SMCs), and macrophages, which play an important role in the formation and development of atherosclerosis (Surmi and Hasty, 2010). Inflammatory diseases like atherosclerosis are greatly influenced by immune system dysregulation and excessive chemokine production. Atherosclerosis prevention and treatment may benefit from the suppression of chemokine-chemokine receptors (Noels et al., 2019).

### CXCL1/CXCR2

LDL is deposited under the endothelium and oxidized, and one of its components, hemolytic phosphatidic acid, can stimulate ECs to release CXCL1 chemokinetin by binding to hemolytic phosphatidic acid one and hemolytic phosphatidic acid three receptors in the endometrium. Serum levels rose and bound to CXCR2 receptors to recruit monocytes and neutrophils to the walls of blood vessels where lipids are deposited (Soehnlein et al., 2013). At the end of a 16-week high-fat diet, the deficiency of the CXCL1 receptor CXCR2 in the Ldlr^−/−^ mice’s bone marrow showed a significant reduction in atherosclerotic lesions and a decrease in the formation of lesioned macrophages, indicating a pro-atherosclerotic role for leukocyte-specific CXCR2 (Boisvert et al., 1998). One study found that injection of CXCL1 neutralizing antibody reduced the number of macrophages and plaque formation at the lesion. This may be achieved by inhibiting the initial macrophage activity mentioned above.

### CCL2/CCR2

Strong inflammatory chemokine CCL2, also known as MCP-1, communicates through its receptor CCR2. Several cell types, including monocytes, macrophages, smooth muscle cells, and endothelial cells in atherosclerotic plaques, produce the chemokine CCL2. CCL2 acts as a trigger for monocyte trafficking across the endothelium during the onset and development of atherosclerosis. One of the earliest stages of atherosclerosis is thought to occur when it binds to the CCR2 receptor expressed by monocytes and directs these cells to move to the subendothelial region.

Numerous investigations conducted on animals have shown that CCL2 levels are correlated with an increased risk of atherosclerosis. According to the study, mice lacking the LDL receptor and MCP-1 had 83% less lipid build up in their aortas. The presence of fewer macrophages in the aortic walls of MCP-1^−/−^/LDL^−/−^mice was consistent with MCP-1’s monocyte chemoattractant characteristics (Gu et al., 1998). Another animal study also showed that selective deletion of CCR2 significantly reduced lesion formation in ApoE^−/−^ mice (Boring et al., 1998). The CCL2-CCR2 axis is a potent driver of atherosclerosis. Compared with CCR2^+/+^/ApoE^−/−^ mice, the area of the neointimal plaque decreased by 47% in CCR2^−/−^/ApoE^−/−^ mice. In addition, CCR2 deletion increased the SMC content and reduced the neointimal macrophage content significantly, validating the function of the CCL2/CCR2 axis in early monocyte recruitment to injured arteries (Schober et al., 2004). The phase 2 human trial of another study showed that blocking CCR2 with MLN1202, a highly specific humanized monoclonal antibody that prevents CCL2 binding, decreased the CRP level in patients at risk for CVD, and it is still a promising target for future treatments (Gilbert et al., 2011).

### CXCL12/CXCR4

CXCL12, also known as stromal cell-derived factor 1α (SDF-1α), is an endostatin, inflammatory dual-use chemokine with chemotactic effects on endothelial precursor cells, monocytes, macrophages, and B and T lymphocytes (Pavlasova et al., 2016). CXCR4 is the receptor for CXCL12 and expressed in most tissues and organs of the body. In 2007, the study of Genome-Wide Association Analysis identified CXCL12 as one of the important and unique candidate genes for CVD (Zeller et al., 2017).

Local injection of CXCL12 into mice with ischemic hindlimbs demonstrated that CXCL12 increased *in vivo* EC progenitor recruitment in ischemic tissues, suggesting that locally administration of SDF1 could increase vasculogenesis and subsequently aid in ischemic neovascularization *in vivo* by increasing recruitment of endothelial progenitor cell (EPCs) in ischemic tissues (Yamaguchi et al., 2003). Additionally, CXCR4 in ECs prevents the development of atherosclerosis by maintaining the function of the endothelial barrier, whereas CXCR4 in SMCs keeps normal phenotype and contractility of the cells. Another study showed that inhibition of the CXCL12/CXCR4 axis with CXCR4 inhibitors partially blocked the homing and migration of EPCs to the lesion site (Abbott et al., 2004). Despite the FDA’s 2008 approval of the CXCR4 antagonist Plerixafor (AMD3100) for mobilizing hematopoietic stem cells, no clinical trials examining the impact of CXCR4/CXCL12 interference about CVD have been published. Thus, CXCL12/CXCR4 axis may become a new target for clinical treatment of CHD (Döring et al., 2014).

## Vaccines against atherosclerosis

### ApoB100

Atherosclerosis is often accompanied with an autoimmune response to LDL and other antigens that can exacerbate or ameliorate the disease progression. Studies have shown that induction of protective immunity with LDL or ApoB peptides in animal models could prevent atherosclerosis. Subcutaneous administration of LDL to rabbits has been shown to reduce atherosclerosis lesions (Gero et al., 1959). Subsequent studies have demonstrated the protective effect of different LDL preparations in different species of animals immunized against atherosclerosis (Palinski et al., 1995).

Retained LDL is susceptible to oxidation, which produces a variety of oxidized forms of LDL including LDL that has been changed by malondialdehyde (Hansson and Hermansson, 2011). The study investigated the protective effect of the natural immune response to malondialdehyde modified LDL (MDA-LDL) in atherosclerosis caused by Porphyromonas gingivalis, a major periodontal disease pathogen. Prior to topical application challenge with live *P. gingivalis*, Ldlr^−/−^ mice were inoculated with mouse MDA-LDL without adjuvant. Comparatively to mice that only received a P. gingivalis challenge, MDA-LDL immunization reduced aortic lipid depositions after challenge. Ldlr^−/−^ mice were immunized with homologous MDA-LDL, which stimulated the production of IL-5 and suggested widespread activation of B-1 cells (Turunen et al., 2015). Another study also confirmed that MDA-LDL neoantigen immuno-atherosclerotic mice had atherosclerotic protective effects (Martos-Folgado et al., 2022).

Knowing that LDL level elevation plays a crucial role in the etiology of atherosclerosis, researchers have extensively explored the impact of antibodies that target the primary protein component of LDL (ApoB100) to eliminate this proatherogenic component. Several studies on LDL or ApoB-100-related vaccines have shown that atherosclerosis vaccines have good atheroprotective effects in animal models (Nilsson et al., 2013; Dunér et al., 2021). P2, p45, p143 and p210 in ApoB-100-related peptides have good immunogenicity, of which p210 is the most studied and most promising epitope (Fredrikson et al., 2003a; Fredrikson et al., 2003b). Among them, p210 is the most studied and most promising epitope, and preclinical safety evaluation trials with the related vaccines are under way (Boisvert et al., 1998). Several experiments have shown that the p210 vaccine is effective in reducing the incidence of atherosclerosis, but the mechanism by which the vaccine acts against atherosclerosis is not fully understood. Studies have found that both humoral immunity (B cells) and cellular immunity (T cells) play an important role (Fredrikson et al., 2008; Klingenberg et al., 2010).

In addition to antibody-mediated vaccines, tolerogenic vaccines are gradually showing good efficacy. In the case of tolerogenic vaccinations, the treatment is designed to enlarge the population of Tregs cells to down regulate the inflammatory reaction numerous self-antigens cause. The current findings reveal that vaccination of ApoE^−/−^ mice with the ApoB peptide vaccine p210 is related with activation of Tregs. Administration of antibodies against CD25 leads in depletion of Tregs and inhibition of the atheroprotective effect of the vaccination. Modulation in atherosclerosis-related autoimmunity by antigen-specific activation of Tregs represents a novel approach for treatment of atherosclerosis (Wigren et al., 2011).

### Heat shock proteins

Heat shock proteins (HSPs) are highly conserved stress proteins present in all cells under normal circumstances and may be produced at high levels in response to stressful situations. HSPs represent another class of autoantigens associated with atherosclerosis. *Mycobacterium* TB HSP65 (mHSP65) and chlamydial HSP60 are highly like human HSP60, and therefore the body’s immunological response to microbial HSPs may cross-react with that of HSPs produced in human vascular cells as a result of stress (Khandia et al., 2017). Jing et al. used oral Lactococcus lactis-delivered mycobacterial HSP65 to immunize Ldlr^−/−^ mice and found that the two oral recombinant L. lactis strains both prevented the HSP65-specific proliferation of bulk splenocytes, and at the same time increased IL-10 production and decreased the interferon-gamma (IFN-γ) level. This groundbreaking study offers a chance for recombinant L. lactis to transport HSP65 to the intestinal mucosa and offers a fresh method for preventing atherosclerosis (Jing et al., 2011). Grundtman et al. reported that ApoE^−/−^ animals fed with chow or high-fat diet were able to tolerate HSP60 or HSP60 peptides when they were administered either orally or intravenously. The lesion size was significantly decreased, and there were also significant quantities of the anti-inflammatory cytokine (IL-10) and an increase in Tregs in the spleen. Accordingly, Potential options for the creation of a tolerizing vaccine to prevent and treat atherosclerosis include atheroprotective mbHSP65 peptides. This can be achieved by constructing vaccines that target anti-atherosclerotic sites while maintaining the functional integrity of the protected area (Grundtman et al., 2015).

### Cholesteryl ester transport protein (CETP)

CETP is a key player in lipid metabolism and a key target for the treatment of atherosclerosis. It mediates the exchange of plasma cholesterylesters (CE) and triacylglycerols (TG) between high-density lipoproteins (HDL) and apolipoprotein B-containing lipoproteins. An abnormal elevation of its activity or content decreases plasma HDL-C concentrations and simultaneously increases LDL-C and VLDL-C concentrations, causing disturbances in lipid metabolism and thus contributing to the development and progression of atherosclerosis (Shrestha et al., 2018). Inhibition of the CETP activity or level can alter the exchange of cholesterol between lipoproteins (HDL and LDL, VLDL), thus exerting an anti-atherogenic effect. Subcutaneous vaccination of rabbits on a high-fat diet with CETi-1 produced antibodies that specifically bound CETP and inhibited its activity, while immunized rabbits showed a 42% increase in plasma HDL level, a 24% decrease in LDL level, and atherosclerotic lesions were reduced by 39.6% in immunized rabbits compared with controls (Rittershaus et al., 2000). However, the phase I clinical trial of CETP vaccine showed that HDL cholesterol levels were not significantly different compared to the blank group (Davidson et al., 2003). Torcetrapib acts as an inhibitor of CETP. A randomized, double-blind clinical study of torcetrapib in patients with high-risk cardiovascular disease reported in 2007. The study enrolled 15,067 high-cardiovascular risk patients who were divided into atorvastatin alone and atorvastatin in combination with torcetrapib. After 12 months of follow-up, HDL was seen to increase by 72.1% and LDL by 24.9%, but the number of deaths was significantly higher in the group receiving torcetrapib and atorvastatin than in the atorvastatin alone group. Torcetrapib did appear to have an off-target impact, but we cannot rule out negative consequences from its suppression of CETP (Barter et al., 2007).

### PCSK9

PCSK9, also known as neural-apoptosis-regulated convertase 1 (NARC-1), is a secreted serine protease. Research showed that PCSK9 acted mainly through the degradation of LDLR. Under normal conditions, LDLR binds LDL particles to form LDL/LDLR complexes, and then enters cells *via* lattice-mediated endocytosis. After LDL degradation, LDLR is recycled to the cell membrane and continues to function (Brown and Ahmed, 2019). PCSK9 in plasma binds to LDL receptors on the surface of hepatocytes, and PCSK9-LDLR conjugates endocytosis into liver cells and is degraded on lysosomes, reducing hepatocytes surface LDLR. Decreased LDLR prevents LDL-C in plasma from entering the liver for metabolism, leading to increased plasma LDL-C levels. The first developed PCSK9 inhibitors are two fully humanized monoclonal antibodies (mAbs), alirocumab and evolocumab. Although monoclonal antibodies have been shown to significantly reduce LDLc, the mAbs face functional limitations due to their relatively short *in vivo* half-life and the need for frequent dosing.

The study of PCSK9 vaccine is still at the stage of animal experimentation. The vaccines that can induce the production of the vaccines that induce the production of PCSK9 antibodies are AT04A and PCSK9Qβ-003. Compared with control, AT04A vaccine induced high and persistent antibody levels against PCSK9, resulting in significant reductions in plasma total cholesterol, LDLc, and inflammatory markers. The atherosclerotic lesion area and aortic inflammation and more lesion-free aortic segments was reduced in AT04A mice compared to control mice (Landlinger et al., 2017). PCSK9Qβ-003 has the same efficacy by decreasing LDL-C levels in mice, inhibited the local inflammatory response, reduced the atherosclerotic area, and slowed down the progression of atherosclerosis (Wu et al., 2021). Clinical development of vaccines faces greater difficulties and longer development cycle, but it could be a breakthrough if the vaccine is effective in human clinical trials. PCSK9 vaccine may provide a cutting-edge therapeutic strategy for the prevention and treatment of cardiovascular illnesses.

## Immune checkpoint

The contact between different immune and non-immune cells is made possible by immune checkpoint proteins, which may either help or hinder immune cell activation (Chen and Flies, 2013). The control of inflammatory reactions depends heavily on this relationship. Immune checkpoint proteins, such as co-inhibitory and co-stimulatory proteins, are known to either enhance or hinder immune cell activation when T cells and antigen-presenting cells interact. APCs and T cells generate immune checkpoint proteins, which give the second signal necessary for cellular activation following the first T cell receptor-mediated activation (Chen and Flies, 2013). Soluble factors like cytokines, which immunological checkpoint proteins also help to regulate in part, serve as the third signal for T cell activation.

Using immune checkpoint proteins to block downstream signaling pathways, nanoparticle technology for cell-type-specific immune checkpoint inhibition, and peptide-drug or antibody-drug conjugates are the main new approaches to combat immune checkpoints. With the widespread use of immune checkpoint inhibitors in cancer therapy, the survival of cancer patients has been effectively prolonged, but the accompanying cardiotoxicity problems have become increasingly prominent, among which atherosclerotic vascular events such as CAD, myocardial infarction and ischemic stroke have attracted the attention of scholars because of their rapid onset and poor prognosis (Pardoll, 2012). Either the B7 or TNF superfamilies are the home to most immunological checkpoint proteins. CD28, CD80,86, CTLA-4 and PD-1, members of the B7 superfamily, are interesting therapeutic targets because they play a significant role in controlling T-cell activity. CD70, CD 27 and CD137 are members of the TNF superfamily. Also included in the T-cell immunoglobulin and mucin domain (TIM) family are strong stimulatory and inhibitory checkpoint proteins.

### CD80/86-CD28/CTLA4

The immunological checkpoint proteins CD80/86-CD28/CTLA4 are essential regulators of atherosclerosis that either promote or suppress plaque inflammation. APCs are the major cells that express CD80 and CD86. Both proteins can interact with the co-stimulatory protein CD28, and the co-inhibitory molecule CTLA4. In contrast to CD28 activation, which increases T cell activation, survival, and memory cell formation in both CD4 and CD8 T cells, CTLA4 activation restricts T cell activation and triggers regulatory T cell responses, thus reducing inflammation and preventing autoimmunity. Macrophages express CD80, CD86 and CD28 in human atherosclerosis lesions. It was shown that the expression of CD80 was increased in CD86 monocyte derived DCs from CAD patients as compared with that in healthy controls (Dopheide et al., 2007).

Atherosclerosis was ameliorated and the production of IFN-c in the lymph nodes and spleen was reduced by their effector T cells in both CD80 and CD86-deficient hyperlipidemic animals. A study showed that CD80 and CD86 controlled the growth of atherosclerosis lesions and the activation of T cells that recognize lesional antigens (Buono et al., 2004). Another study demonstrated that CTLA-4 overexpression dramatically decreased the development of atherosclerosis and the intraplaque concentration of macrophage and CD4 (^+^) T cells in the aortic root as compared with controls (Matsumoto et al., 2016). All these data show that CTLA4 inhibited plaque development by generating anti-inflammatory T cell responses, whereas CD80/86-CD28 co-stimulation promoted atherosclerosis by eliciting Th1 responses. Rheumatoid arthritis can be treated with abatacept, a CTLA4-Ig construct that blocks CD80/CD86 from interacting with CD28 and activating T cells (Blair and Deeks, 2017). Clinical trials have not evaluated cardiovascular parameters, despite preclinical research’ suggestions of positive effects on CVD (Ma et al., 2013). Therefore, prospective treatments for AS include stimulating CTLA4 or inhibiting CD80/86-CD28 by medication.

### CD40-CD40L/TRAF6

The CD40L-CD40 dyad is another effective immune-checkpoint target for atherosclerosis, which controls a variety of immunological activities, including T cell activation, immunoglobulin isotype switching, macrophage, DC and B-cell activation and proliferation (Kusters et al., 2018). Most immune cells in the circulation as well as immune and non-immune cells within the atherosclerotic plaque express CD40 and CD40L during the development of atherosclerosis (Mach et al., 1997). In Ldlr^−/−^mice, antibody-mediated suppression of CD40L or CD40 lowered the burden of AS plaques and produced collagen-rich plaques with few immune cells, which is the murine analogue of a stable plaque (Schönbeck et al., 2000). Leukocyte recruitment to the areas of vascular inflammation is facilitated by CD40-CD40L interactions during the early stages of plaque development. Lipid deposition and foam cell production are boosted by soluble CD40 ligand markedly, which is also believed to be related to the increased expression of CD36 and scavenger receptor type A. By reducing CD36 expression on macrophages, the absence of CD40 also lowered foam cell production, which may further restrain the growth of AS lesions (Yuan et al., 2015). Although blocking the relationship between CD40 and CD40L is a possible treatment for atherosclerosis, blocking CD40L may not be a therapeutically feasible option because long-term blockade impairs the immune response throughout the body. TNF receptor-associated factors (TRAFs) are adaptor molecules required for CD40 to exert signaling, for they lack intrinsic signaling properties. According to the findings, CD40-TRAF6 interactions, which predominately occur in macrophages, are particularly important in driving atherosclerosis (Lutgens et al., 2010).

A small-molecule inhibitor (SMI) protects CD40-mediated immunity by blocking the interaction between CD40 and TRAF6 while leaving CD40-TRAF2/3/5 interactions unaffected. TRAF-STOP therapy with either SMI 6860766 or 6877002 decreased the onset of early atherosclerosis and stopped the progression of established atherosclerosis in ApoE^−/−^ mice (Seijkens et al., 2018). Immune checkpoint therapies for atherosclerosis will be practical and secure when they involve highly selective immune checkpoint modulation through interfering with cell-specific downstream signaling pathways or tailored medication delivery.

## Targeting the adaptive immune system

A growing body of research indicates that Tregs, which are in charge of preserving immunological tolerance and squelching excessive immune responses, may play a critical role in halting the onset and progression of atherosclerosis by controlling pathogenic immunoinflammatory responses (Sasaki et al., 2015). Treg homeostasis *in vivo* depends on IL-2 (Littman and Rudensky, 2010). Local delivery of IL-2 to atherosclerotic lesions in ApoE^−/−^ mice or administration of IL-2/anti-IL-2 mAb immunocomplexes result in a decrease in atherosclerosis because Treg expansion. The first double-blind, placebo-controlled phase I/II clinical trial, LILACS, is currently underway and aims to evaluate the safety and effectiveness of low-dose IL-2 in patients with stable ischemic heart disease and in patients with ACS (Zhao et al., 2018). Meanwhile, in patients with acute coronary syndromes, the IVORY study will examine how low-dose IL-2 affects ACS (Sriranjan et al., 2022). It remains to be seen whether these clinical studies will have the desired results.

Directly attacking atherogenic B cell subsets is another strategy for suppressing adaptive immune cells. Multiple sclerosis and RA are already being treated clinically using B cell depletion therapy, and studies in mice have demonstrated that selective B2 cell depletion with the use of an anti-CD20 antibody decreases atherosclerosis (Ait-Oufella et al., 2010; Pattarabanjird et al., 2021). Rituximab in patients with acute ST-elevation myocardial infarction (RITA-MI) was a prospective, open-label, dosage-escalation, single-arm, phase 1/2a clinical study testing rituximab given as a single intravenous dose in patients with STEMI within 48 h of symptom onset. Safety was the first goal; subsequent objectives included changes in circulating immune cell subsets, particularly B cells, as well as cardiac and inflammatory indicators. The study shows that when administered in the context of acute STEMI, a single infusion of rituximab looks safe and significantly modifies circulating B-cell subsets (Zhao et al., 2022).

## Conclusion

**FIGURE 3:**
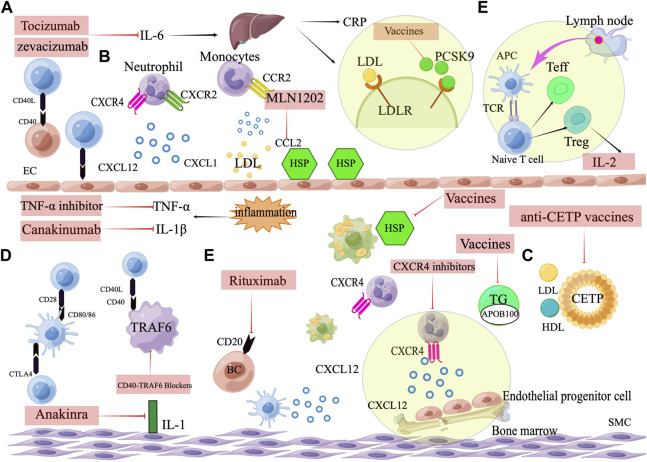
Targets for immunotherapy of atherosclerosis. **(A)** Pro-inflammatory cytokine inhibitors, including IL-1β inhibitors (Canakinumab), IL-1 inhibitors (Anakinra), IL-6 inhibitors (Tocilizumab, Zevacizumab), and TNF-α inhibitors. **(B)** Chemokines and their receptors. Neutrophil cells containing the CXCR2 receptor arrest and move on the endothelium when the endothelium is activated and expresses the chemokine CXCL1. Monocyte that express CCR2 are attracted and adhered to CCL2. CXCL12, when combined with CXCR4, promotes the proliferation and migration of EPCs in the bone marrow and can keep the phenotype of SMCs normal and contractile. **(C)** Vaccines against atherosclerosis, including ApoB100 vaccines, heat shock protein vaccines, CETP vaccines and PCSK9 vaccines. **(D)** Immune checkpoint. Activation of T cells by costimulation and leucocyte adherence are two methods through which CD40-CD40 ligand interactions promote atherosclerosis. CD80/CD86 can interact with the co-stimulatory protein CD28, and the co-inhibitory molecule CTLA4. CTLA4 inhibits plaque progression by generating anti-inflammatory T cell responses, whereas CD80/86-CD28 co-stimulation promotes atherosclerosis by eliciting Th1 responses. The interaction between CD40 and TRAF6 in macrophages is what triggers atherosclerosis and macrophage activation.**(E)**Targeting the adaptive immune system. Therapeutic targeting adaptive immunity include low dose IL-2 targeting Treg cells and B cell depletion therapy. Rituximab targets a marker of B-cells (CD20).

The intrinsic immune response, mainly monocyte-macrophage, as well as the adaptive immune response induced by T cells and B cells, constitutes a complex immune network in atherosclerosis. Animal experiments and clinical data have demonstrated the involvement of immune response in atherosclerosis formation and progression. Therefore, it is a promising strategy to prevent and treat atherosclerosis by regulating immune suppression of intraplaque inflammation and reducing intraplaque autoimmune response. However, many challenges remain in the translation of immunotherapy from animal studies to clinical studies, such as the safety and stability of vaccines, the effective duration of immunization, the effective cut-off point of clinical trials, and the impact on existing atherosclerosis therapeutic measures. It is expected that more immunotherapeutic strategies will be used for the treatment of ASCVD, which will further improve the theory of atherosclerosis and the prognosis of CVD patients (Figure 3).
